# Employment status in cancer patients the first five years after diagnosis—a register-based study

**DOI:** 10.1007/s11764-024-01576-5

**Published:** 2024-04-08

**Authors:** E. Brink, M. S. Pilegaard, T. G. Bonnesen, C. V. Nielsen, P. Pedersen

**Affiliations:** 1 Department of Public Health, Aarhus University, Aarhus, Denmark; 2 DEFACTUM, Central Denmark Region, Aarhus, Denmark; 3 Department of Social Medicine and Rehabilitation, Goedstrup Hospital, Herning, Denmark

**Keywords:** Labor market, Employment, Vocational rehabilitation, Matched controls, Cohort study

## Abstract

**Purpose:**

Work is important for identity formation, social status, and economic independency. Although some evidence within the field of work and cancer survivorship exists, no study has so far investigated employment status across all cancer diagnoses. Thus, the aim of the present study was to investigate the impact of all cancer diagnoses on employment status.

**Methods:**

Danish cancer patients aged 20–60 years, diagnosed between 2000 and 2015, were identified through Danish registers and matched 1:5 with cancer-free controls. Logistic and linear regression was performed separately in 11 cancer types to assess and compare work status and work participation between cancer patients and cancer-free controls one, three, and five years after diagnosis.

**Results:**

A total of 111,770 cancer patients and 507,003 cancer-free controls were included. All cancer types had lower chances of working one year after diagnosis (ORs between 0.05 and 0.76), with lung, colorectal, upper gastrointestinal, and blood cancer patients having the lowest chances. After three years, 10 of 11 cancer types had lower chances (ORs between 0.39 and 0.84). After five years, there were minimal differences between cancer patients and controls among most cancer types (ORs between 0.75 and 1.36).

**Conclusion:**

Most cancer patients had lower chances of working compared with the general population until five years after diagnosis. However, patients with certain cancer types experienced lower chances of working all years, despite improvement over time.

**Implications for Cancer Survivors:**

The knowledge will help increase awareness on challenges regarding work-life after cancer. Furthermore, the distinguishing between diagnoses can inform to more targeted vocational rehabilitation.

**Supplementary Information:**

The online version contains supplementary material available at 10.1007/s11764-024-01576-5.

## Background

Work is for many people seen as an essential part of everyday life and is important for identity formation, social status, and economic independency [1, 2]. In Europe, approximately 40% of all cancers are diagnosed in people of working age [3]. With the continuing improvements in diagnostic tools, screening programs, and cancer treatments, as well as the rising retirement ages, the number of cancer patients, who are still part of the work force, will increase in the next few years [4]. Thus, there will be an increasing need for rehabilitation to maintain cancer patients in employment.

Evidence shows that returning to work after a cancer diagnosis is associated with improved self-esteem, general and mental health, and better quality of life [5, 6]. Most cancer patients are strongly motivated to return to work as they consider this a sign of complete recovery [7]. Despite this, cancer patients are at increased risk of temporarily or permanently losing employment [8–11]. A systematic review found an average rate of return to work after cancer of 64%. The estimate was based on rates of return to work that ranged from 24 to 94% between the included studies [12]. Another review found similar results with estimates of return to work ranging between 39 and 77% [13]. The wide ranges could be due to varying definitions of outcome, pooling of studies investigating different cancer diagnoses, hereof mostly breast cancer, and differences in health care and social security systems between countries [10, 14]. Hence, the estimate of 64% does not represent the overall rate for all cancers nor does it differentiate between the separate cancer diagnoses.

Although considerable amount of evidence exists on the field of employment and cancer survivorship, no newly register-based study has, to our knowledge, investigated employment status in all cancer diagnoses [15, 16]. Recent studies have focused on a single type of cancer or included only a limited number of diagnoses, typically the most prevalent types like breast, colorectal, and prostate cancer [17–19]. This leads to an elevated risk of overlooking other types of cancers with a high incidence of unemployment or disability pension. By focusing on all types of cancers, it is possible to identify the impact of a specific cancer diagnosis on the risk of losing employment. This knowledge can inform healthcare professionals and contribute to future development of vocational rehabilitation for cancer patients.

The present study is aimed at examining the association between cancer and employment status in the first five years after diagnosis in all cancer patients compared to a population of matched, cancer-free controls.

## Methods

### Study design

The present study is a historical cohort study using Danish registers to access data on all incident cancer patients in Denmark.

### Data sources

The study was based on data from several Danish population-based registries: Statistics Denmark (STD), the Danish Cancer Register (CAR) [20], the Danish National Patient Register (DNPR) [21], and the Danish Register for Evaluation of Marginalization (DREAM) [22]. CAR contains information on all incident cancers in Denmark since 1943. DNPR contains information on all somatic inpatient visits since 1977 and all outpatient, as well as psychiatric visits since 1995. DREAM provides weekly information on time periods in which Danish citizens receive social benefits, like unemployment benefits, sickness benefits, and disability pension. DREAM has previously been validated in comparison to workplace-registered data on sick leave [23] and self-reported data on type of income [22] and was found to have good validity. The unique personal identity number, which is provided to each Danish citizen, enabled the linkage of data across these registries.

### Study population

In the present study, data from an already existing cohort were used, which investigated the risk of disability pension following cancer compared to a population of matched cancer-free persons [24]. All persons aged 20–60 years diagnosed with a first-time diagnosis of cancer in the period from January 2000 to December 2015 were identified in CAR along with the date of diagnosis. Only cancer diagnoses categorized according to Nordic Cancer statistics (NORDCAN), a database on all cancers registered in the Nordic countries, were included [25]. All non-melanoma skin cancers were excluded due to the heterogeneous and incomplete classification of the diagnosis [26]. Persons with multiple cancer diagnoses on the same date were also excluded. Each cancer patient was matched with five controls identified through STD. Matching was performed on gender, age (10-year-age strata), highest completed education (primary/high school, vocational education, education < 3 years, bachelor degree, or master degree), and household income in Euros (< − 60,395, − 60,394 – − 20,132, − 20,131 – − 1, 0 − 20,131, 20,132 – 40,263, 40,264 – 60,394, and > 60,395), defined at the time of diagnosis of the cancer patient. The controls were included at the same date of diagnosis as their matched cancer patient. Thus, this date is defined as baseline. Controls were considered ineligible if they had been diagnosed with cancer previously to baseline. To ensure this, the personal identity number from CAR was used to identify and exclude all individuals in STD, who had been diagnosed with cancer prior to this date. Any person who received disability pension at baseline or had missing information on education or income was excluded from the study population as well.

### Exposures

The study population was divided into 11 categories based on NORDCAN [25]. The 11 categories of cancers were upper gastrointestinal, colorectal, lung, breast, gynecological, male genitals, kidney and bladder, melanoma skin, central nervous system (CNS), blood, and other sites.

### Outcomes

Two overall outcomes were estimated in order to quantify the impact of a cancer diagnosis on subsequent employment status. The outcomes were:

- Working (yes/no) one, three, and five years after time of diagnosis
- Work participation defined as the number of weeks working during years one, three, and five

Persons were categorized as working if they did not receive any social benefits. Moreover, persons who received unemployment benefits or flexible job compensation were categorized as working, because they were considered either fit for duty but not currently having a job or having a reduced ability to work, i.e., they are available for the labor market.

In order to be considered as working at years one, three, and five, a person had to be in one of the aforementioned categories for four consecutive weeks before the week of measurement. Persons receiving sickness benefits were considered not working, because despite having a job, they are not actively participating in the labor market. In Denmark, sickness benefits are paid by the local authorities. However, the first weeks of sickness absence are paid by the employer and thus do not appear in DREAM. The period of employer-paid sickness absence varied from 14 to 30 days during the inclusion period, and therefore, sickness absence lasting less than 30 days was regarded as working.

### Covariates

The following covariates were included in the present study:

1. Ethnicity identified through DREAM and categorized as Danish, Western (non-Danish), and non-Western
2. Episodes of sick leave from 12 to 24 months prior to time of diagnosis identified in DREAM. A washout period of 12 months prior to diagnosis was chosen, because sick leave in this period could be explained by the undiagnosed cancer
3. Comorbidity status in the five years prior to diagnosis/inclusion date using the Charlson comorbidity index (CCI) scored on a 3-point severity scale. The total sum of scores was used to divide the comorbidity index into three categories of 0, 1–2, and ≥ 3. Data on 19 somatic comorbidities from the DNPR were used to generate comorbidity status

The selection of the three covariates was informed by previous research indicating a possible influence of these factors on employment outcomes after cancer. Differences in ethnicity have been linked to varying rates of return to work, while a history of comorbidities or previous episodes of unemployment or sick leave has been shown to negatively impact employment outcomes [18, 27–29].

### Statistical analyses

All analyses and descriptive statistics were performed separately for each cancer type. Number and percent were used to describe the 11 cancer types regarding the matching variables and potential confounders. Comparison of work status between cancer patients and controls was estimated using logistic regression models. The estimates are presented as odds ratios (ORs) with 95% confidence intervals (CI) for years one, three, and five. Furthermore, the number and percentage of cancer patients and controls working are presented for each year. The mean work participation, in number of weeks, was calculated for both cancer patients and controls, and linear regression models were applied to estimate the mean differences between the groups during years one, three, and five [30]. For both outcome measures, analyses were made as crude models, one model adjusted for matching variables and another model adjusted for matching variables in addition to ethnicity and sick leave 12–24 months prior to diagnosis. However, the estimates were similar in the crude model and the model adjusted for matching variables. Thus, only the model adjusted for matching variables in addition to sick leave and ethnicity is presented in the results. All persons who had permanently left the labor market at the end of each follow-up year, i.e., went on disability pension, old-age pension, emigrated, or died were excluded from the analyses. The total number of and reasons for exclusion are presented for years one, three, and five. STATA version 17.0 was used as statistical software [31].

### Ethics

The study was approved by the Danish Data Protection Agency (no. 1–16-02–445-16), and all personal identifiers were removed from the dataset by STD.

## Results

### Study population

**Fig. 1 increase font in table:**
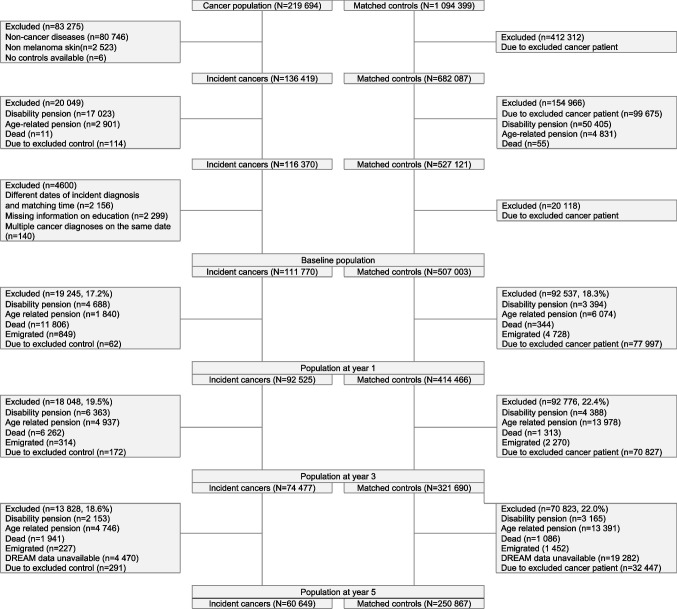
Flow chart of the exclusion process from initial to final study population

A total of 219,694 cancer patients aged between 20 and 60 years were from 2000 to 2015 identified in CAR. The controls included 1,094,399 cancer-free persons (Fig. 1). Of these, 111,770 cancer patients and 507,003 controls were eligible for the study and thus represent the study population. The main reason for exclusion among the cancer patients were (1) non-cancer diseases or non-melanoma skin cancer (*n* = 83,269) and (2) permanent withdrawal from the labor market at baseline (*n* = 19,935). The controls who were matched with the ineligible cancer patients were also excluded. Furthermore, the study population at each year of follow-up varied due to exclusion of cancer patients who died, emigrated, and retired during follow-up (Fig. 1).

### Baseline characteristics

The most prevalent cancer types were breast (21.1%), upper gastrointestinal (10.7%), and melanoma skin cancer (10.7%) (Table 1). Overall, most cancer patients were Danish and did not have any comorbidities. About 69% had primary, high school, or vocational education as their highest attained education, and 58% were in the age group of 50–60 years. Lung, colorectal, and kidney and bladder cancer patients were generally older, while gynecological, melanoma skin, male genital, and CNS cancer patients were younger, with between 25.3% and 33.6% being in the age group of 20–39 (Table 1). Patients with breast and melanoma skin cancer were the highest educated, where > 33% had a bachelor’s degree or long further education. In contrast, lung, kidney and bladder, and upper gastrointestinal cancer patients were less well educated, with ≤ 20% having a bachelor’s degree or long further education. Moreover, they were the cancer patients with the highest number of weeks on sick leave prior to diagnosis (Table 1).

**Table 1:** Baseline characteristics of the study population

		NORDCAN group										
	All cancers	Upper gastro-intestinal	Colorectal	Lung	Breast	Gynecological	Male genitals	Kidney and bladder	Melanoma skin	CNS	Blood	Other
	*n* (%)	*n* (%)	*n* (%)	*n* (%)	*n* (%)	*n* (%)	*n* (%)	*n* (%)	*n* (%)	*n* (%)	*n* (%)	*n* (%)
All	111,770 (100)	11,967 (10.7)	9957 (8.9)	8359 (7.5)	23,549 (21.1)	8218 (7.4)	9905 (8.9)	6404 (5.7)	11,913 (10.7)	8167 (7.3)	8069 (7.2)	5262 (4.7)
Age												
20–29	5658 (5.06)	322 (2.7)	113 (1.1)	41 (0.5)	216 (0.9)	569 (6.9)	1032 (10.4)	79 (1.2)	1357 (11.4)	797 (9.8)	751 (9.3)	381 (7.2)
30–39	13,124 (11.7)	956 (8.0)	478 (4.8)	217 (2.6)	2182 (9.3)	1509 (18.4)	1506 (15.2)	340 (5.3)	2640 (22.2)	1516 (18.6)	1080 (13.4)	700 (13.3)
40–49	28,193 (25.2)	2885 (24.1)	2038 (20.5)	1504 (18.0)	7638 (32.4)	2146 (26.1)	1345 (13.6)	1387 (21.7)	3614 (30.3)	2375 (29.1)	1923 (23.8)	1338 (25.4)
50–60	64,795 (58.0)	7804 (65.2)	7328 (73.6)	6597 (78.9)	13,513 (57.4)	3994 (48.6)	6022 (60.8)	4598 (71.8)	4302 (36.1)	3479 (42.6)	4315 (53.5)	2843 (54.0)
Gender												
Female	62,403 (55.8)	4187 (35.0)	4398 (44.2)	3856 (46.1)	23,463 (99.6)	8218 (100.0)	0 (0.0)	1618 (25.3)	7018 (58.9)	4186 (51.3)	3077 (38.1)	2382 (54.3)
Male	49,367 (44.2)	7780 (65.0)	5559 (55.8)	4503 (53.9)	86 (0.4)	0 (0.0)	9905 (100.0)	4786 (74.7)	4895 (41.1)	3981 (48.7)	4992 (61.9)	2880 (54.7)
Highest attained education												
Primary and high school	32,232 (28.8)	3868 (32.3)	2842 (28.5)	3250 (38.9)	6085 (25.8)	2528 (30,8)	2538 (25.6)	1927 (30.1)	2719 (22.8)	2339 (28.6)	2511 (31.1)	1625 (30.9)
Vocational education	45,217 (40.5)	5209 (43.5)	4294 (43.1)	3647 (43.6)	8633 (36.7)	3029 (36.9)	4456 (45.0)	2946 (46.0)	4510 (37.9)	3210 (39.3)	3161 (39.2)	2122 (40.3)
Short further education	5295 (4.7))	502 (4.2)	497 (5.0)	253 (3.0)	1097 (4.7)	324 (3.9)	570 (5.8)	332 (5.2)	696 (5.8)	427 (5.2)	374 (4.6)	223 (4.2)
Bachelor’s degree	20,770 (18.6)	1743 (14.6)	1650 (16.6)	940 (11.3)	5874 (24.9)	1815 (22.1)	1396 (14.1)	837 (13.1)	2733 (22.9)	1501 (18.4)	1357 (16.8)	924 (17.6)
Long further education	8256 (7.4)	645 (5.4)	674 (6.8)	269 (3.2)	1860 (7.9)	522 (6.4)	945 (9.5)	362 (5.7)	1255 (10.5)	690 (8.5)	666 (8.3)	368 (7.0)
Income in euros												
< − 1*	1763 (1.6)	363 (3.0)	154 (1.6)	342 (4.1)	167 (0.7)	88 (1.1)	115 (1.2)	91 (1.4)	88 (0.7)	114 (1.4)	110 (1.4)	131 (2.5)
0–20,131	28,669 (25.7)	3908 (32.7)	2201 (22.1)	3072 (36.8)	5339 (22.7)	2485 (30.2)	1840 (18.6)	1521 (23.8)	2304 (19.3)	2201 (27.0)	2182 (27.0)	1616 (30.7)
20,132–40,263	64,034 (57.3)	6191 (51.7)	5841 (58.7)	4194 (50.2)	14,982 (63.6)	4910 (59.8)	5407 (54.6)	3704 (57.8)	7015 (58.9)	4545 (55.7)	4444 (55.1)	2801 (53.2)
40,264–60,394	13,198 (11.8)	1169 (9.8)	1336 (13.4)	590 (7.1)	2542 (10.8)	624 (7.6)	1795 (18.1)	805 (12.6)	1858 (15.6)	972 (11.9)	983 (12.2)	524 (10.0)
> 60,394	4106 (3.7)	336 (2.8)	425 (4.3)	161 (1.9)	519 (2.2)	111 (1.4)	748 (7.6)	283 (4.4)	648 (5.4)	335 (4.1)	350 (4.3)	190 (3.6)
Ethnicity												
Danish	103,839 (92.9)	10,892 (91.0)	9336 (93.8)	7706 (92.2)	21,838 (92.7)	7619 (92.7)	9419 (95.1)	5927 (92.6)	11,484 (96.4)	7459 (91.3)	7348 (91.1)	4811 (91.4)
Western	3019 (2.7)	339 (2.8)	245 (2.5)	213 (2.5)	695 (3.0)	244 (3.0)	243 (2.5)	151 (2.4)	280 (2.4)	221 (2.7)	256 (3.2)	132 (2.5)
Non-western	3660 (3.3)	546 (4.6)	267 (2.7)	242 (2.9)	778 (3.3)	254 (3.1)	175 (1.8)	259 (4.0)	97 (0.8)	436 (5.3)	380 (4.7)	226 (4.3)
Unknown	1252 (1.1)	190 (1.6)	109 (1.1)	198 (2.4)	238 (1.0)	101 (1.2)	68 (0.7)	67 (1.0)	52 (0.4)	51 (0.6)	85 (1.1)	93 (1.8)
Comorbidity 5 years before												
0	103,004 (92.2)	10,500 (87.7)	9217 (92.6)	7350 (87.9)	22,328 (94.8)	7758 (94.4)	9219 (93.1)	5734 (89.5)	11,333 (95.1)	7546 (92.4)	7346 (91.0)	4673 (88.8)
1–2	7770 (7.0)	1211 (10.1)	654 (6.6)	911 (10.9)	1144 (4.9)	420 (5.1)	613 (6.2)	589 (9.2)	525 (4.4)	577 (7.1)	615 (7.6)	511 (9.7)
3 +	996 (0.9)	256 (2.1)	86 (0.9)	98 (1.2)	77 (0.3)	40 (0.5)	73 (0.7)	81 (1.3)	55 (0.5)	44 (0.5)	108 (1.3)	78 (1.5)
Sick leave 12–24 months before diagnosis (weeks)												
0	93,928 (84.0)	9784 (81.8)	8451 (84.9)	6715 (80.3)	19,836 (84.2)	6865 (83.5)	8599 (86.8)	5305 (82.8)	10,427 (87.5)	6779 (83.0)	6836 (84.7)	4331 (82.3)
1–7	9996 (9.0)	1170 (9.8)	844 (8.5)	893 (10.7)	2052 (8.7)	776 (9.4)	787 (8.0)	589 (9.2)	867 (7.3)	794 (9.7)	703 (8.7)	521 (9.9)
8–27	5574 (5.0)	705 (5.9)	482 (4.8)	515 (6.2)	1176 (5.0)	412 (5.0)	384 (3.9)	372 (5.8)	438 (3.7)	417 (5.1)	388 (4.8)	285 (5.4)
≥ 28	2272 (2.0)	308 (2.6)	180 (1.8)	236 (2.8)	485 (2.1)	165 (2.0)	135 (1.4)	138 (2.2)	181 (1.5)	177 (2.2)	142 (1.8)	125 (2.4)

^*^Income groups: <  − 60,395, -60394 – -20,132, and -20,131 – -1 have been categorized as one group due to a small number of observations

### Work status

Overall, cancer patients worked less often than controls; 63.4% of cancer patients and 88.4% of controls worked at year one (Table 2). At year three, 83.5% and 88.5% were working, respectively, and at year five, the numbers were comparable for cancer patients (88.4%) and controls (88.8%). When looking at each cancer type, the chances of working among cancer patients compared with controls were lower for all cancer types the first year post-diagnosis with ORs ranging from 0.05 to 0.76 (Table 2). The lowest chances were seen in lung, upper gastrointestinal, colorectal, and blood cancer patients of whom 40–60% were working. At year three, the chances had improved but were still lower for ten cancer types (ORs between 0.39 and 0.84). The lowest chances were still seen in the four aforementioned cancer types, though the percentages of cancer patients working in these groups had increased to 78.7–83.6%. Only melanoma skin cancer patients had higher chances of working compared with controls (OR = 1.14 [1.07–1.22]), with 88% of cancer patients working. Five years post-diagnosis, the chances of working were similar for cancer patients and controls in most cancer types. However, in seven cancer types, it was still slightly lower (ORs between 0.75 and 0.89). For kidney and bladder, gynecological, male genital, and melanoma skin cancer patients, the chances of working were equal or higher compared with controls (ORs between 0.91 and 1.36).

**Table 2:** Work status among cancer patients and controls and odds ratios (OR) for cancer patients working in each NORDCAN group one, three, and five years after time of diagnosis

		Cancer patients	Controls	Crude^A^	Adjusted model^B^
		Working, *n* (%)	Working, *n* (%)	OR (95%-CI)	OR (95%-CI)
All					
	Year 1	58,632 (63.4)	366,276 (88.4)	0.23 (0.22, 0,23)	0.18 (0.18, 0.19)
	Year 3	62,216 (83.5)	284,643 (88.5)	0.66 (0.65, 0.68)	0.63 (0.62, 0.65)
	Year 5	53,609 (88.4)	222,721 (88.8)	0.96 (0.94, 0.99)	0.96 (0.93, 0.99)
Upper gastrointestinal					
	Year 1	4068 (53.6)	29,347 (88.1)	0.16 (0.15, 0.16)	0.12 (0.11, 0.13)
	Year 3	4199 (79.8)	19,649 (88.3)	0.52 (0.48, 0.57)	0.49 (0.45, 0.53)
	Year 5	3580 (87.1)	14,671 (88.5)	0.88 (0.79, 0.97)	0.87 (0.78, 0.97)
Colorectal					
	Year 1	4891 (59.2)	33,358 (91.3)	0.14 (0.13, 0.15)	0.11 (0.11, 0.12)
	Year 3	5050 (83.6)	23,307 (91.4)	0.48 (0.44, 0.59)	0.46 (0.42, 0.50)
	Year 5	4007 (89.4)	16,294 (91.3)	0.80 (0.72, 0.89)	0.81 (0.72, 0.90)
Lung					
	Year 1	1322 (40.0)	12,741 (90.2)	0.07 (0.07, 0.08)	0.05 (0.04, 0.05)
	Year 3	1118 (80.2)	4953 (89.0)	0.50 (0.43, 0.59)	0.39 (0.33, 0.46)
	Year 5	873 (89.17)	3252 (89.3)	0.98 (0.78, 1.23)	0.75 (0.59, 0.95)
Breast					
	Year 1	12,535 (56.2)	87,997 (89.2)	0.16 (0.15, 0.16)	0.13 (0.12, 0.13)
	Year 3	15,908 (83.6)	72,265 (89.5)	0.60 (0.57, 0.63)	0.58 (0.55, 0.60)
	Year 5	13,780 (88.4)	56,492 (89.6)	0.88 (0.83, 0.93)	0.87 (0.82, 0.92)
Gynecological					
	Year 1	4778 (64.7)	27,796 (84.8)	0.33 (0.31, 0.35)	0.27 (0.26, 0.29)
	Year 3	4729 (80.0)	21,518 (84.9)	0.71 (0.66, 0.77)	0.66 (0.61, 0.71)
	Year 5	4148 (85.6)	17,205 (85.8)	0.98 (0.90, 1.08)	0.94 (0.85, 1.03)
Male genitals					
	Year 1	7364 (79.7)	38,522 (90.6)	0.41 (0.38, 0.43)	0.35 (0.32, 0.37)
	Year 3	7090 (88.9)	31,992 (90.6)	0.83 (0.77, 0.90)	0.84 (0.77, 0.92)
	Year 5	5904 (91.8)	24,707 (90.6)	1.17 (1.06, 1.29)	1.22 (1.10, 1.35)
Kidney and bladder					
	Year 1	4010 (74.5)	21,512 (90.8)	0.30 (0.27, 0.32)	0.28 (0.25, 0.30)
	Year 3	3686 (86.1)	16,265 (91.0)	0.61 (0.55, 0.68)	0.65 (0.58, 0.72)
	Year 5	3028 (89.0)	12,109 (90.7)	0.83 (0.74, 0.94)	0.91 (0.80, 1.03)
Melanoma skin					
	Year 1	9578 (83.7)	45,889 (86.4)	0.80 (0.76, 0.85)	0.76 (0.72, 0.81)
	Year 3	9392 (88.0)	41,601 (86.6)	1.13 (1.06, 1.21)	1.14 (1.07, 1.22)
	Year 5	8398 (90.4)	35,421 (87.6)	1.34 (1.24, 1.44)	1.36 (1.25, 1.47)
CNS					
	Year 1	3901 (57.5)	26,543 (85.8)	0.22 (0.21, 0.24)	0.17 (0.16, 0.19)
	Year 3	4194 (79.4)	20,158 (86.0)	0.62 (0.58, 0.67)	0.60 (0.55, 0.65)
	Year 5	3797 (85.5)	16,606 (87.2)	0.87 (0.79, 0.95)	0.89 (0.81, 0.99)
Blood					
	Year 1	3784 (54.2)	27,395 (87.4)	0.17 (0.16, 0.18)	0.12 (0.11, 0.13)
	Year 3	4415 (78.7)	21,380 (87.6)	0.52 (0.49, 0.56)	0.48 (0.45, 0.53)
	Year 5	3940 (86.0)	16,781 (87.7)	0.86 (0.78, 0.95)	0.87 (0.79, 0.96)
Other					
	Year 1	2401 (62.3)	15,176 (86.9)	0.25 (0.23, 0.27)	0.19 (0.17, 0.21)
	Year 3	2435 (80.8)	11,555 (87.7)	0.59 (0.53, 0.66)	0.55 (0.49, 0.61)
	Year 5	2154 (86.2)	9183 (88.0)	0.86 (0.75, 0.97)	0.85 (0.74, 0.97)

^a^Unadjusted. ^b^Adjusted for matching variables as well as ethnicity, and sick leave 12–24 months before baseline

### Work participation

During the first year, cancer patients worked an average of 28.0 weeks per year, while controls worked an average of 47.5 weeks per year (Table 3). For controls, the mean work participation remained steady during follow-up. For cancer patients, it increased significantly during the third year (44.8 weeks), and by year five, the average weeks were comparable between cancer patients (47.4 weeks) and controls (47.7 weeks). When looking at each cancer type, the differences in mean work participation during the first year were most pronounced for lung and breast cancer patients, followed by colorectal, upper gastrointestinal, and blood cancer patients who worked on average between 23.2 and 32.4 weeks less than their controls. During the third year, lung cancer patients still had the largest difference in work participation, with an average of 6.6 weeks less than their controls, followed by blood, upper gastrointestinal, and colorectal cancer patients who worked between 4.0 and 4.7 weeks less compared with their controls (Table 3). In contrast, male genital cancer and melanoma skin cancer patients had the lowest difference during the third year, working on average 0.9 weeks less and 0.7 weeks more than their controls, respectively. During the fifth year after diagnosis, the differences were even further reduced and most cancer patients worked < 1.5 week less than their controls. Male genital and melanoma skin cancer patients worked on average 0.6 and 1.3 weeks more than their controls.

**Table 3:** Mean work participation (WP) measured in weeks per year and the differences in mean WP between cancer patients and controls in each NORDCAN group the first, third, and fifth year after time of diagnosis

		Cancer patients	Controls	Crude^A^	Adjusted model^B^
		Mean WP cancer patients	Mean WP controls	Difference in mean WP	Difference in mean WP
		Weeks (95%-CI)	Weeks (95%-CI)	Weeks (95%-CI)	Weeks (95%-CI)
All					
	Year 1	28.0 (27.8, 28.1)	47.5 (47.4, 47.5)	− 19.5 (− 19.6, − 19.4)	− 19.5 (− 19.6, − 19.4)
	Year 3	44.8 (44.7, 44.9)	47.5 (47.4, 47.5)	− 2.6 (− 2.8, − 2.5)	− 2.6 (− 2.7, − 2.5)
	Year 5	47.4 (47.3, 47.5)	47.7 (47.6, 47.7)	− 0.2 (− 0.4, − 0.1)	− 0.2 (− 0.3, − 0.1)
Upper gastrointestinal					
	Year 1	23.1 (22.6, 23.5)	47.3 (47.1, 47.5)	− 24.2 (− 24.6, − 23.8)	− 24.4 (− 24.8, − 24.1)
	Year 3	43.0 (42.5, 43.5)	47.4 (47.2, 47.6)	− 4.4 (− 4.8, − 3.9)	− 4.3 (− 4.7, − 3.9)
	Year 5	46.9 (46.4, 47.3)	47.6 (47.3, 47.8)	− 0.7 (− 1.1, − 0.2)	− 0.6 (− 1.1, − 0.2)
Colorectal					
	Year 1	23.4 (22.9, 23.8)	49.0 (48.9, 49.1)	− 25.6 (− 26.0, − 25.3)	− 25.7 (− 26.0, − 25.4)
	Year 3	44.8 (44.4, 45.3)	49.0 (48.9, 49.2)	− 4.2 (− 4.5, − 3.8)	− 4.0 (− 4.4, − 3.7)
	Year 5	48.2 (47.8, 48.6)	49.0 (48.8, 49.2)	− 0.8 (− 1.2, − 0.4)	− 0.8 (− 1.1, − 0.4)
Lung					
	Year 1	17.9 (17.2, 18.6)	49.0 (48.3, 48.7)	− 30.6 (− 31.2, − 30.1)	− 32.4 (− 32.9, − 31.9)
	Year 3	43.1 (42.2, 44.0)	48.1 (47.8, 48.5)	− 5.1 (− 5.9, − 4.2)	− 6.6 (− 7.4, − 5.7)
	Year 5	47.7 (46.8, 48.6)	48.2 (47.8, 48.6)	− 0.5 (− 1.4, 0.5)	− 1.8 (− 2.8, − 0.8)
Breast					
	Year 1	19.6 (19.4, 19.9)	47.9 (47.8, 48.0)	− 28.3 (− 28.5, − 28.1)	− 28.6 (− 28.8, − 28.4)
	Year 3	45.0 (44.8, 45.2)	48.0 (47.9, 48.1)	− 3.0 (− 3.2, − 2.8)	− 3.0 (− 3.2, − 2.8)
	Year 5	47.6 (47.4, 47.8)	48.1 (48.0, 48.2)	− 0.5 (− 0.7, − 0.2)	− 0.5 (− 0.7, − 0.3)
Gynecological					
	Year 1	27.5 (27.0, 28.0)	45.6 (45.5, 45.8)	− 18.1 (− 18.5, − 17.7)	− 18.5 (− 18.9, − 18.1)
	Year 3	43.0 (42.6, 43.5)	45.6 (45.4, 45.8)	− 2.5 (− 3.0, − 2.1)	− 2.8 (− 3.2, − 2.3)
	Year 5	45.9 (45.5, 46.3)	45.8 (45.6, 46.1)	0.1 (− 0.4, 0.6)	− 0.2 (− 0.7, 0.3)
Male genitals					
	Year 1	39.3 (39.0, 39.7)	48.6 (48.4, 48.7)	− 9.3 (− 9.6, − 8.9)	− 9.1 (− 9.3, − 8.8)
	Year 3	47.5 (47.2, 47.8)	48.5 (48.4, 48.7)	− 1.0 (− 1.3, − 0.7)	− 0.9 (− 1.1, − 0.6)
	Year 5	49.0 (48.7, 49.3)	48.6 (48.4, 48.7)	0.4 (0.1, 0.8)	0.6 (0.3, 0.9)
Kidney & bladder					
	Year 1	36.9 (36.3, 37.4)	48.7 (48.6, 48.9)	− 11.9 (− 12.3, − 11.5)	− 11.4 (− 11.8, − 11.0)
	Year 3	46.2 (45.7, 46.6)	48.8 (48.6, 49.0)	− 2.6 (− 3.1, − 2.2)	− 2.1 (− 2.5, − 1.7)
	Year 5	48.0 (47.6, 48.4)	48.9 (48.7, 49.1)	− 0.9 (− 1.4, − 0.5)	− 0.5 (− 1.0, − 0.1)
Melanoma skin					
	Year 1	43.2 (42.9, 43.5)	46.3 (46.2, 46.4)	− 3.1 (− 3.4, − 2.8)	− 3.1 (− 3.4, − 2.8)
	Year 3	47.1 (46.8, 47.3)	46.4 (46.3, 46.6)	0.7 (0.3, 1.0)	0.7 (0.4, 0.9)
	Year 5	48.3 (48.0, 48.5)	46.9 (46.8, 47.1)	1.4 (1.0, 1.7)	1.3 (1.0,.1.6)
CNS					
	Year 1	26.8 (26.3, 27.4)	46.1 (46.0, 46.3)	− 19.3 (− 19.7, − 18.8)	− 19.0 (− 19.4, − 18.6)
	Year 3	42.3 (41.8, 42.8)	46.1 (45.9, 46.3)	− 3.8 (− 4.3, − 3.3)	− 3.5 (− 4.0, − 3.1)
	Year 5	45.7 (45.3, 46.2)	46.8 (46.6, 47.0)	− 1.1 (− 1.5, − 0.6)	− 0.8 (− 1.3, − 0.4)
Blood					
	Year 1	23.6 (23.1, 24.2)	46.9 (46.8, 47.1)	− 23.3 (− 23.7, − 22.9)	− 23.2 (− 23.6, − 22.8)
	Year 3	42.1 (41.6, 42.6)	47.0 (46.8, 47.1)	− 4.9 (− 5.3, − 4.4)	− 4.7 (− 5.1, − 4.3)
	Year 5	45.9 (45.4, 46.3)	47.2 (47.0, 47.4)	− 1.3 (− 1.8, − 0.8)	− 1.1 (− 1.6, − 0.7)
Other					
	Year 1	30.1 (29.4, 30.8)	46.8 (46.6, 47.1)	− 16.7 (− 17.3, − 16.2)	− 16.9 (− 17.4, − 16.4)
	Year 3	43.5 (42.8, 44.1)	47.0 (46.7, 47.2)	− 3.5 (− 4.1, − 2.9)	− 3.5 (− 4.1, − 3.0)
	Year 5	46.1 (45.5, 46.7)	47.3 (47.0, 47.5)	− 1.2 (− 1.8, − 0.5)	− 1.2 (− 1.8, − 0.6)

^a^Unadjusted. ^b^Adjusted for matching variables as well as ethnicity and sick leave 12–24 months before baseline

## Discussion

The present historical cohort study investigated work status and work participation within the first five years after diagnosis among cancer patients in eleven different cancer types compared to populations of matched cancer-free controls. The findings show that, across all cancer types, cancer patients had a lower chance of working and a lower work participation compared with cancer-free controls during and after the first year. By the third year, the prospects had improved for all, and after five years, there were minimal differences between cancer patients and controls. However, employment prospects varied between the different cancer types, and after five years, seven cancer types still had both a lower chance of working and a lower work participation compared to their cancer-free controls.

Previous studies have found an increased risk of unemployment, sickness absence, and disability pension among cancer patients [15, 32, 33]. However, no previous study has investigated all cancer diagnoses separately to the extent as our study. Our findings show that lung, colorectal, upper gastrointestinal, and blood cancer patients had the lowest chances of working at all years. In contrast, kidney and bladder, gynecological, male genital, and melanoma skin cancer patients seemed to have equal or higher chances of working compared with their matched controls after five years. While extensive research has been made on the impact of colorectal cancer on work, indicating an increased risk of experiencing adverse work outcomes [34, 35], less research has been conducted on the impact of upper gastrointestinal cancer. Our results show that cancer patients of this type had one of the lowest chances of working at all-time points. Despite the limited number of previous studies, a negative impact on unemployment and return to work has been found in some diagnoses of the gastrointestinal cancer type [36, 37]. Thus, our findings add to the evidence of gastrointestinal cancer, although more research is needed. Regarding lung cancer, a systematic review [38] found that lung cancer patients had an increased risk of unemployment and sick leave compared to persons with non-malignant chronic diseases, other cancer types, or cancer-free persons. According to our results, lung cancer patients had the lowest chance of working of all cancer types at all-time points. This is further supported by several studies, which found that lung cancer patients had the highest risk of unemployment and the longest time to return to work among several cancer diagnoses [32, 39]. Besides lung cancer, blood, colorectal, and gastrointestinal cancer patients have been found to have the highest risk of unemployment or sick leave [15, 40]. Hence, our results align with previous research in the understanding of which cancer types have the highest risk of adverse work outcomes, adding further value to the evaluation of employment status after cancer.

Several studies have indicated that factors such as age, income, educational level, and ethnicity are associated with employment status after cancer [32, 33, 41, 42]. When examining the baseline characteristics of the study population, it reveals an uneven distribution of these factors across the different cancer types. Lung, colorectal, and upper gastrointestinal cancer patients tended to be older, less educated, and had lower incomes compared to the overall cancer population (Table 1). Thus, these factors could partially account for the lower chances of working among patients with these cancer types. Conversely, we found that melanoma skin cancer patients had a higher chance of working compared with controls after three and five years, consistent with previous research [12]. Cancer patients with melanoma were generally younger, and higher educated than the overall cancer population, which, combined with the low mortality of the disease [43], likely contributes to the good work prognosis for this particular cancer type. On the other hand, blood cancer patients experienced a low chance of working at all years, despite being younger and having a similar educational level compared to the overall cancer population. Moreover, kidney and bladder cancer patients were older and less educated than the overall population but still had similar odds of working as their controls after five years. Thus, other factors are likely to have contributed to the causality of employment status. Other factors that have been identified in previous research as associated with work outcomes include treatment modalities, symptom burden, type of work, and physical and mental comorbidities [16, 27, 44, 45]. However, information on the majority of these factors were not available in our data.

Besides the influence on employment, it is well-established knowledge that socioeconomic status impacts the incidence and mortality of cancer [46]. For many, particularly tobacco- and lifestyle-associated cancers, the incidence and mortality is higher among people who have low education, low income, and are living alone. We excluded all who died, emigrated, and received disability or age-related pension during follow-up, as we wanted to investigate the patients who survived their cancer and were available for employment. This resulted in considerable reductions in the study population over time, and we therefore explored potential differences in population characteristics between baseline and follow-up. This was presented in two post hoc descriptive tables, showing the characteristics along with the reasons for exclusions after the third and fifth years (Supplementary Table 1 and 2). Most exclusions during follow-up were due to death or disability pension of which the highest numbers were seen in lung, colorectal upper gastrointestinal, and CNS cancer. For all cancer types, the population available at both years three and five had a higher proportion of females, were younger and higher educated, had higher income, had no comorbidities, and had no sick leave before diagnosis, compared with the population at baseline. This suggests that cancer patients, who were more socioeconomically advantaged at baseline, had higher chances of remaining available for employment after cancer. Notably, the previously discussed variations in baseline characteristics between the different cancer types showed a similar pattern. The cancer types consisting of a higher proportion of patients with low socioeconomic status, such as lung, colorectal, and upper gastrointestinal cancer, were also the cancer types with the lowest chances of working. Taken together, these observations indicate a possible association between low socioeconomic status and poor employment prospects. However, we cannot conclude whether the differences between the baseline population and the follow-up samples are merely a result of higher mortality among those with lowest socioeconomic status. Neither can we conclude whether the distribution of these characteristics between working and not working cancer patients follows the same tendency. Despite this, our results showed that certain cancer types had poorer employment prospects than others, and it is likely that the observed differences in employment status between the eleven cancer types are not solely attributable to the specific cancer type but rather are a result of complex interactions between several socioeconomic, demographic, and diagnosis-specific factors. Thus, it is important to consider all these factors in the organization and implementation of vocational rehabilitation for cancer patients.

Only few interventions, directly aimed at vocational aspects of rehabilitation for cancer patients, have been evaluated, and the evaluations of such interventions have shown ambiguous effects [47, 48]. Furthermore, a population-based study found that socioeconomically disadvantaged cancer patients reported a higher need for rehabilitation but were to a lesser extent participating in rehabilitation services [49]. This supports our theory of social inequity in employment prospects after cancer. Therefore, more research on the subject is needed in order to identify cancer patients who potentially could benefit from vocational rehabilitation and to enhance their participation in such interventions.

Another aspect to consider when evaluating employment status after cancer is the time perspective. Our results showed that all cancer types had lower chances of working after the first year compared with cancer-free controls. However, for most cancer types, odds of working were similar as for controls after five years. Most previous studies found comparable time perspectives, despite some variations. One study found the highest rate of sick leave during the first year [33]. Another study found that 90% of all cancer patients who returned to work did so within the first two years [50], and further two studies found the highest risk of unemployment between two and four years after diagnosis [15, 51]. The differences in peaking risk might be the result of varying outcome definitions. Irrespective, the overall evidence suggest that the risk of experiencing adverse work outcomes is highest during the first years post-diagnosis, after which the risk decreases and stabilizes at a level either equal to or higher than that of cancer-free controls. The negative impact of cancer treatment on the ability to work [44] could possibly explain this. Although treatment duration varies with type and extent of the cancer, some cancer patients are likely to still receive treatment or be in the recovery phase one year after diagnosis [52]. In order to achieve the greatest effect of vocational rehabilitation, interventions should therefore be initiated early in the disease trajectory to support patients in resuming work once they have finished treatment.

### Strengths and limitations

A strength of the present study is the high validity of data and complete follow-up using several well-documented Danish registers and the ability to cross-link them with the unique personal identification number given to all Danish citizens. Another strength of the study is the large study population of all Danish cancer patients, and the inclusion of a matched control group, which, together with the minimal amount of missing information, decreased the risk of selection and information bias. However, given the large sample size, minor differences in odds of working between cancer patients and controls were likely to result in statistically significant estimates, which must be considered in the interpretation of the clinical importance of the results.

We adjusted for several important factors in the matching process and during analyses. However, we were unable to adjust for disease-specific factors like stage of the cancer disease or type of treatment [42]. Furthermore, we lacked information on a number of psychosocial and work-related factors like cohabitation, mental illness, and type of work [16, 19, 41]. Collectively, the inability to adjust for these important factors could therefore have confounded the results.

We only included persons who were still alive and available for employment at each year of follow-up. Thus, all persons who had died or received disability pension or age-related pension in between each follow-up were excluded from the subsequent analyses. This has possibly led to an underestimation of the overall impact of cancer on employment status due to healthy worker bias. However, we wished to investigate employment status among cancer patients who could potentially benefit from vocational rehabilitation initiatives in order to investigate the need for such.

## Conclusion

This register-based historical cohort study shows that all cancer patients had lower chances of working compared to cancer-free controls one year after diagnosis. Despite that all cancer patients experienced increasing chances of working up to five years after diagnosis, and many to a level comparable to the general population, some cancer types still had lower chances of working after five years. Of these, the lowest chances were seen in lung, colorectal, upper gastrointestinal, and blood cancer patients. Thus, increasing awareness must be paid to patients with these diagnoses to reintegrate them into employment. Furthermore, our results highlight the need for more research to explore the influence of socioeconomic factors on employment status after cancer. This research would allow a more specific identification of cancer patients who could benefit from vocational rehabilitation.

## Supplementary Information

Below is the link to the electronic supplementary material.

Supplementary file1 (DOCX 29 KB)

Supplementary file2 (DOCX 25 KB)

## Data Availability

Data not available due to legal restrictions as data is stored on a server at Statistics Denmark.
