# Supplementary material for: Employment status in cancer patients the first five years after diagnosis—a register-based study
Source: J Cancer Surviv. 2024 Apr 8;19(5):1598–610. doi: 10.1007/s11764-024-01576-5 (PMC12460457; doi:10.1007/s11764-024-01576-5)
Supplement: Supplementary file 1 — Supplementary file1 (DOCX 29 KB) [file 11764_2024_1576_MOESM1_ESM.docx]

| **Supplementary Table 1:**Descriptive characteristics of the study population at the end of 3 years of follow-up. | | | | | | | | | | | | | |
| --- | --- | --- | --- | --- | --- | --- | --- | --- | --- | --- | --- | --- | --- |
|  |  |  | NORDCAN group | | | | | | | | | | |
|  | All cancers |  | Upper gastro-intestinal | Colorectal | Lung | Breast | Gynaeco-logical | Male genitals | Kidney & bladder | Melanoma skin | CNS | Blood | Other |
|  | n (%) |  | n (%) | n (%) | n (%) | n (%) | n (%) | n (%) | n (%) | n (%) | n (%) | n (%) | n (%) |
| All | 74,477 (100) |  | 5,262 (7,1) | 6,044 (8.1) | 1,394 (1.9) | 19,027 (25.6) | 5,909 (7.9) | 7,977 (10.7) | 4,282 (5.8) | 10,674 (14.3) | 5,284 (7.1) | 5,611 (7.5) | 3,013 (4.1) |
| Age |  |  |  |  |  |  |  |  |  |  |  |  |  |
| 20-29 | 4,874 (6.5) |  | 265 (5.0) | 74 (1.2) | 20 (1.4) | 170 (0.9) | 491 (8.3) | 963 (12.1) | 69 (1.6) | 1,271 (11.9) | 644 (12.2) | 635 (11.3) | 272 (9.0) |
| 30-39 | 11,087 (14.9) |  | 701 (13.3) | 325 (5.4) | 65 (4.7) | 1902 (10.0) | 1,330 (22.5) | 1,431 (17.9) | 288 (6.7) | 2,495 (23.4) | 1,191 (22.5) | 859 (15.3) | 500 (16.6) |
| 40-49 | 21,562 (29.0) |  | 1,515 (28.8) | 1,421 (23.5) | 298 (21.4) | 6879 (36.2) | 1,674 (28.3) | 1,240 (15.6) | 1,056 (24.7) | 3,395 (31.8) | 1,670 (31.6) | 1,506 (26.8) | 908 (30.1) |
| 50-60 | 36,954 (49.6) |  | 2,781 (52.9) | 4,224 (69.9) | 1,011 (72.5) | 10,076 (53.0) | 2,414 (40.9) | 4,343 (54.4) | 2,869 (67.0) | 3,513 (32.9) | 1,779 (33.7) | 2,611 (46.5) | 1,333 (44.2) |
| Gender |  |  |  |  |  |  |  |  |  |  |  |  |  |
| Female | 44,356 (59.6) |  | 2,243 (42.6) | 2,686 (44.4) | 721 (51.7) | 18,959 (99.6) | 5,909 (100.0) | 0 (0.0) | 1,047 (24.5) | 6,391 (59.9) | 2,883 (54.6) | 2,099 (37.4) | 1,418 (47.1) |
| Male | 30,121 (40.4) |  | 3,019 (57.4) | 3,358 (55.6) | 673 (48.3) | 68 (0.4) | 0 (0.0) | 7,977 (100.0) | 3,235 (75.6) | 4,283 (40.1) | 2,401 (45.4) | 3,512 (62.6) | 1,595 (52.9) |
| Highest attained education |  |  |  |  |  |  |  |  |  |  |  |  |  |
| Primary & high school | 19,233 (25.8) |  | 1,507 (28.6) | 1,585 (26.2) | 527 (37.8) | 4,509 (23.7) | 1,690 (28.6) | 1,969 (24.7) | 1,207 (28.2) | 2,373 (22.2) | 1,411 (26.7) | 1,634 (29.1) | 821 (27.3) |
| Vocational education | 29,165 (39.2) |  | 2,212 (42.1) | 2,544 (42.1) | 564 (40.5) | 6,874 (36.1) | 2,174 (36.8) | 3,541 (44.4) | 1,929 (45.1) | 3,993 (37.4) | 2,013 (38.1) | 2,132 (38.0) | 1,188 (39.4) |
| Short further education | 3,986 (5.4) |  | 262 (5.0) | 344 (5.7) | 44 (3.2) | 939 (4.9) | 250 (4.2) | 491 (6.2) | 260 (6.1) | 639 (6.0) | 308 (5.8) | 281 (5.0) | 168 (5.6) |
| Bachelor's degree | 15,488 (20.8) |  | 924 (17.6) | 1,096 (18.1) | 186 (13.3) | 5,016 (26.4) | 1,374 (23.3) | 1,140 (14.3) | 607 (14.2) | 2,504 (23.5) | 1,032 (19.5) | 1,011 (18.0) | 598 (19.9) |
| Long further education | 6,605 (8.9) |  | 356 (6.8) | 475 (7.9) | 73 (5.2) | 1,689 (8.9) | 421 (7.1) | 836 (10.5) | 279 (6.5) | 1,165 (10.9) | 520 (9.8) | 553 (9.9) | 238 (7.9) |
| Income in euros |  |  |  |  |  |  |  |  |  |  |  |  |  |
| < -1* | 714 (1.0) |  | 82 (1.6) | 65 (1.1) | 61 (4.4) | 128 (0.7) | 42 (0.7) | 81 (1.0) | 48 (1.1) | 72 (0.7) | 48 (0.8) | 47 (0.8) | 40 (1.3) |
| 0 – 20,131 | 14,899 (20.0) |  | 1,189 (22.6) | 983 (16.3) | 376 (27.0) | 3,637 (19.1) | 1,559 (26.4) | 1,350 (16.9) | 792 (18.5) | 1,914 (17.9) | 1,190 (22.5) | 1,271 (22.7) | 638 (21.2) |
| 20,132 - 40,263 | 45,028 (60.5) |  | 3,095 (58.8) | 3,650 (60.4) | 752 (54.0) | 12,463 (65.5) | 3,703 (62.7) | 4,315 (54.1) | 2,612 (61.0) | 6,339 (59.4) | 3,098 (58.6) | 3,201 (57.1) | 1,800 (59.7) |
| 40,264 – 60,394 | 10,481 (14.1) |  | 697 (13.3) | 1,014 (16.8) | 162 (11.6) | 2,316 (12.2) | 510 (8.6) | 1,550 (19.4) | 608 (14.2) | 1,741 (16.3) | 698 (13.2) | 797 (14.2) | 388 (12.9) |
| > 60,394 | 3,355 (4.5) |  | 199 (3.8) | 332 (5.5) | 43 (3.1) | 483 (2.5) | 95 (1.6) | 681 (8.5) | 222 (5.2) | 608 (5.7) | 250 (4.7) | 295 (5.3) | 147 (4.9) |
| Ethnicity |  |  |  |  |  |  |  |  |  |  |  |  |  |
| Danish | 68,962 (92.6) |  | 4,637 (88.1) | 45,621 (93.0) | 1,125 (80.7) | 17,637 (92.7) | 5,491 (92.9) | 7,599 (95.3) | 3,921 (91.6) | 10,305 (96.5) | 4,805 (90.9) | 5,101 (90.9) | 2,720 (90.3) |
| Western | 1,971 (2.6) |  | 146 (2.8) | 160 (2.6) | 38 (2.7) | 552 (2.9) | 162 (2.7) | 180 (2.3) | 106 (2.5) | 234 (2.2) | 134 (2.5) | 181 (3.2) | 78 (2.6) |
| Non-western | 2,309 (3.1) |  | 291 (5.5) | 155 (2.6) | 39 (2.8) | 602 (3.2) | 157 (2.7) | 130 (1.6) | 188 (4.4) | 85 (0.8) | 294 (5.6) | 245 (4.4) | 123 (4.1) |
| Unknown | 1,235 (1.7) |  | 188 (3.6) | 108 (1.8) | 192 (13.8) | 236 (1.2) | 99 (1.7) | 68 (0.9) | 67 (1.6) | 50 (0.5) | 51 (1.0) | 84 (1.5) | 92 (3.1) |
| Comorbidity 5 years before |  |  |  |  |  |  |  |  |  |  |  |  |  |
| 0 | 69,927 (93.9) |  | 4,820 (91.6) | 5,649 (93.5) | 1,216 (87.2) | 18,184 (95.6) | 5,631 (95.3) | 7,488 (93.9) | 3,884 (90.7) | 10,185 (95.4) | 4,950 (93.7) | 5180 (92.3) | 2,740 (90.9) |
| 1-2 | 4,139 (5.6) |  | 378 (7.2) | 360 (6.0) | 166 (11.9) | 798 (4.2) | 255 (4.3) | 447 (5.6) | 357 (8.3) | 448 (4.2) | 313 (5.9) | 370 (6.6) | 247 (8.2) |
| 3+ | 411 (0.6) |  | 64 (1.2) | 35 (0.6) | 12 (0.9) | 45 (0.2) | 23 (0.4) | 42 (0.5) | 41 (1.0) | 41 (0.4) | 21 (0.4) | 61 (1.1) | 26 (0.9) |
| Sick leave 12-24 months before diagnosis (weeks) |  |  |  |  |  |  |  |  |  |  |  |  |  |
| 0 | 63,957 (85.9) |  | 4,447 (84.5) | 5,236 (86.6) | 1,152 (82.6) | 16,285 (85.6) | 4,961 (84.0) | 7,013 (87.9) | 3,615 (84.4) | 9,395 (88.0) | 4,458 (84.4) | 4,840 (86.3) | 2,555 (84.8) |
| 1-7 | 6,231 (8.4) |  | 486 (9.2) | 463 (7.7) | 146 (10.5) | 1,570 (8.3) | 575 (9.7) | 599 (7.5) | 385 (9.0) | 772 (7.2) | 495 (9.4) | 467 (8.3) | 273 (9.1) |
| 8-27 | 3,198 (4.3) |  | 257 (4.9) | 261 (4.3) | 72 (5.2) | 866 (4.6) | 273 (4.6) | 283 (3.6) | 216 (5.0) | 364 (3.4) | 242 (4.6) | 233 (4.2) | 131 (4.4) |
| ≥ 28 | 1,091 (1.5) |  | 72 (1.4) | 84 (1.4) | 24 (1.7) | 306 (1.6) | 100 (1.7) | 82 (1.0) | 66 (1.5) | 143 (1.3) | 89 (1.7) | 71 (1.3) | 54 (1.8) |

| **Supplementary Table 1 (continued):** | | | | | | | | | | | | | |
| --- | --- | --- | --- | --- | --- | --- | --- | --- | --- | --- | --- | --- | --- |
|  |  |  | NORDCAN group | | | | | | | | | | |
|  | All cancers |  | Upper gastro- intestinal | Colorectal | Lung | Breast | Gynaeco-logical | Male genitals | Kidney & bladder | Melanoma skin | CNS | Blood | Other |
| Excluded due to: n (%**) |  |  |  |  |  |  |  |  |  |  |  |  |  |
| Disability pension | 11,051 (9.9) |  | 1,784 (14.9) | 1,097 (11.0) | 1,978 (23.7) | 1,469 (6.2) | 768 (9.3) | 485 (4.9) | 544 (8.5) | 213 (1.8) | 1,308 (16.0) | 809 (10.0) | 596 (11.3) |
| Age-related pension | 6,777 (6.1) |  | 481 (4.0) | 818 (8.2) | 293 (3.5) | 1,828 (7.8) | 524 (6.4) | 871 (8.8) | 571 (8.9) | 434 (3.6) | 272 (3.3) | 477 (5.9) | 208 (4.0) |
| Death | 18,068 (16.2) |  | 4,303 (36.0) | 1,921 (19.3) | 4,643 (55.5) | 963 (4.1) | 898 (10.9) | 409 (4.1) | 936 (14.6) | 415 (3.5) | 1,161 (14.2) | 1,048 (13.0) | 1,371 (26.1) |
| Emigration | 1,163 (1.0) |  | 110 (0.9) | 56 (0.6) | 35 (0.4) | 193 (0.8) | 90 (1.1) | 152 (1.5) | 60 (0.9) | 162 (1.4) | 133 (1.6) | 108 (1.3) | 64 (1.2) |

* Income groups: < -60,395, -60394 – -20,132, and -20,131 – -1 have been categorized as one group due to a small number of observations.

** Percentage of total number of cancer patients in each cancer group at baseline.
