# Supplementary material for: Employment status in cancer patients the first five years after diagnosis—a register-based study
Source: J Cancer Surviv. 2024 Apr 8;19(5):1598–610. doi: 10.1007/s11764-024-01576-5 (PMC12460457; doi:10.1007/s11764-024-01576-5)
Supplement: Supplementary file 2 — Supplementary file2 (DOCX 25 KB) [file 11764_2024_1576_MOESM2_ESM.docx]

| **Supplementary Table 2:** Descriptive characteristics of the study population at the end of follow up. | | | | | | | | | | | | | |
| --- | --- | --- | --- | --- | --- | --- | --- | --- | --- | --- | --- | --- | --- |
|  |  |  | NORDCAN group | | | | | | | | | | |
|  | All cancers |  | Upper gastro-intestinal | Colorectal | Lung | Breast | Gynaeco-logical | Male genitals | Kidney & bladder | Melanoma skin | CNS | Blood | Other |
|  | n (%) |  | n (%) | n (%) | n (%) | n (%) | n (%) | n (%) | n (%) | n (%) | n (%) | n (%) | n (%) |
| All | 60,649 (100) |  | 4,112 (6.8) | 4,481 (7.4) | 979 (1.6) | 15,595 (25.7) | 4,846 (8.0) | 6,430 (10.6) | 3,402 (5.6) | 9,286 (15.3) | 4,439 (7.3) | 4,581 (7.6) | 2,498 (4.1) |
| Age |  |  |  |  |  |  |  |  |  |  |  |  |  |
| 20-29 | 4,414 (7.3) |  | 235 (5.7) | 61 (1.4) | 20 (2.0) | 141 (0.9) | 458 (9.5) | 904 (14.1) | 58 (1.7) | 1,179 (12.7) | 543 (12.2) | 572 (12.5) | 243 (9.7) |
| 30-39 | 10,139 (16.7) |  | 639 (15.5) | 279 (6.2) | 58 (5.9) | 1,696 (10.9) | 1,216 (25.1) | 1,356 (21.1) | 258 (7.6) | 2,328 (25.1) | 1,059 (23.9) | 791 (17.3) | 459 (18.4) |
| 40-49 | 19,108 (31.5) |  | 1,303 (31.7) | 1,199 (26.8) | 230 (23.5) | 6,143 (39.4) | 1,443 (29.8) | 1,132 (17.6) | 965 (28.4) | 3,084 (33.2) | 1,482 (33.4) | 1,321 (28.8) | 806 (32.3) |
| 50-60 | 26,988 (44.5) |  | 1,935 (47.1) | 2,942 (65.7) | 671 (68.5) | 7.615 (48.8) | 1,729 (35.7) | 3,038 (47.3) | 2,121 (62.4) | 2,695 (29.0) | 1,355 (30.5) | 1,897 (41.4) | 990 (39.6) |
| Gender |  |  |  |  |  |  |  |  |  |  |  |  |  |
| Female | 36,491 (60.2) |  | 1,861 (45.3) | 1,979 (44.2) | 505 (51.6) | 15,538 (99.6) | 4,846 (100.0) | 0 (0.0) | 817 (24.0) | 5,642 (60.8) | 2,417 (54.5) | 1,705 (37.2) | 1,181 (47.3) |
| Male | 24,158 (39.8) |  | 2,251 (54.7) | 2,502 (55.8) | 474 (48.2) | 57 (0.4) | 0 (0.0) | 6,430 (100.0) | 2,585 (76.0) | 3,644 (39.2) | 2022 (45.6) | 2,876 (62.8) | 1,317 (52.7) |
| Highest attained education |  |  |  |  |  |  |  |  |  |  |  |  |  |
| Primary & high school | 15,485 (25.5) |  | 1,177 (28.6) | 1,139 (25.4) | 372 (38.0) | 3,631 (23.3) | 1,358 (28.0) | 1,615 (25.1) | 941 (27.7) | 2,074 (22.3) | 1,185 (26.7) | 1,322 (28.9) | 671 (26.9) |
| Vocational education | 23,443 (38.7) |  | 1,695 (41.2) | 1,871 (41.8) | 393 (40.1) | 5,557 (35.6) | 1,764 (36.4) | 2,839 (44.2) | 1,529 (45.0) | 3,423 (36.9) | 1,672 (37.7) | 1,731 (37.8) | 969 (38.8) |
| Short further education | 3,332 (5.5) |  | 208 (5.1) | 266 (5.9) | 27 (2.8) | 793 (5.1) | 221 (4.6) | 412 (6.4) | 200 (5.9) | 556 (6.0) | 270 (6.1) | 239 (5.2) | 140 (5.6) |
| Bachelor's degree | 12,816 (21.1) |  | 740 (18.0) | 817 (18.2) | 138 (14.1) | 4,178 (26.8) | 1,141 (23.6) | 891 (13.9) | 494 (14.5) | 2,204 (23.7) | 867 (19.5) | 822 (17.9) | 524 (21.0) |
| Long further education | 5,573 (9.2) |  | 292 (7.1) | 388 (8.7) | 49 (5.0) | 1,436 (9.2) | 362 (7.5) | 673 (10.5) | 238 (7.0) | 1,029 (11.1) | 445 (10.0) | 467 (10.2) | 194 (7.8) |
| Income in euros |  |  |  |  |  |  |  |  |  |  |  |  |  |
| < -1* | 627 (1.0) |  | 74 (1.8) | 55 (1.2) | 58 (5.9) | 115 (0.7) | 36 (0.7) | 67 (1.0) | 40 (1.2) | 67 (0.7) | 41 (0.9) | 38 (0.8) | 36 (1.4) |
| 0 – 20,131 | 11,994 (19.8) |  | 918 (22.3) | 695 (15.5) | 282 (28.8) | 2,844 (18.2) | 1,263 (26.1) | 1,156 (18.0) | 608 (17.9) | 1,670 (18.0) | 969 (21.8) | 1,050 (22.9) | 539 (21.6) |
| 20,132 - 40,263 | 36,976 (61.0) |  | 2,447 (59.5) | 2,721 (60.7) | 496 (50.7) | 10,332 (66.3) | 6,064 (63.2) | 3,494 (54.3) | 2,082 (61.2) | 5,605 (60.4) | 2,644 (59.6) | 2,611 (57.0) | 1,480 (59.3) |
| 40,264 – 60,394 | 8,338 (13.8) |  | 529 (12.9) | 744 (16.6) | 112 (11.4) | 1,915 (12.3) | 409 (8.4) | 1,175 (18.3) | 490 (14.4) | 1,435 (15.5) | 567 (12.8) | 642 (14.0) | 320 (12.8) |
| > 60,394 | 2,714 (4.5) |  | 144 (3.5) | 266 (5.9) | 31 (3.2) | 389 (2.5) | 74 (1.5) | 538 (8.4) | 182 (5.4) | 509 (5.5) | 218 (4.9) | 240 (5.2) | 123 (4.9) |
| Ethnicity |  |  |  |  |  |  |  |  |  |  |  |  |  |
| Danish | 56,082 (92.5) |  | 3,584 (87.2) | 4,154 (92.7) | 745 (76.1) | 14,443 (92.6) | 4,506 (93.0) | 6,130 (95.3) | 3,101 (91.2) | 8,979 (96.7) | 4,045 (91.1) | 4,151 (90.6) | 2,244 (89.8) |
| Western | 1,551 (2.6) |  | 107 (2.6) | 116 (2.6) | 23 (2.3) | 448 (2.9) | 121 (2.5) | 141 (2.2) | 83 (2.4) | 189 (2.0) | 106 (2.4) | 150 (3.3) | 67 (2.7) |
| Non-western | 1,832 (3.0) |  | 238 (5.8) | 108 (2.4) | 24 (2.5) | 477 (3.1) | 128 (2.6) | 94 (1.5) | 152 (4.5) | 69 (0.7) | 241 (5.4) | 199 (4.3) | 102 (4.1) |
| Unknown | 1,184 (2.0) |  | 183 (4.5) | 103 (2.3) | 187 (19.1) | 227 (1.5) | 91 (1.9) | 65 (1.0) | 66 (1.9) | 49 (0.5) | 47 (1.1) | 81 (1.8) | 85 (3.4) |
| Comorbidity 5 years before |  |  |  |  |  |  |  |  |  |  |  |  |  |
| 0 | 57,131 (94.2) |  | 3,782 (92.0) | 4,203 (93.8) | 857 (87.5) | 14,931 (95.7) | 4,628 (95.5) | 6,073 (94.5) | 3,096 (91.0) | 8,872 (95.5) | 4,177 (94.1) | 4,231 (92.4) | 2,281 (91.3) |
| 1-2 | 3,210 (5.3) |  | 290 (7.1) | 253 (5.7) | 113 (11.5) | 632 (4.1) | 199 (4.1) | 323 (5.0) | 279 (8.2) | 381 (4.1) | 246 (5.5) | 297 (6.5) | 197 (7.9) |
| 3+ | 308 (0.5) |  | 40 (1.0) | 25 (0.6) | 9 (0.9) | 32 (0.2) | 19 (0.4) | 34 (0.5) | 27 (0.8) | 33 (0.4) | 16 (0.4) | 53 (1.2) | 20 (0.8) |
| Sick leave 12-24 months before diagnosis (weeks) |  |  |  |  |  |  |  |  |  |  |  |  |  |
| 0 | 52,247 (86.2) |  | 3,480 (84.6) | 3,890 (86.8) | 817 (83.5) | 13,445 (86.2) | 4,076 (84.1) | 5,653 (87.9) | 2,889 (84.9) | 8,168 (88.0) | 3,753 (84.6) | 3,957 (86.4) | 2,119 (84.8) |
| 1-7 | 5,050 (8.3) |  | 382 (9.3) | 333 (7.4) | 98 (10.0) | 1,247 (8.0) | 475 (9.8) | 488 (7.6) | 298 (8.8) | 691 (7.4) | 426 (9.6) | 385 (8.4) | 227 (9.1) |
| 8-27 | 2,508 (4.1) |  | 201 (4.9) | 198 (4.4) | 48 (4.9) | 672 (4.3) | 211 (4.4) | 227 (3.5) | 167 (4.9) | 308 (3.3) | 190 (4.3) | 180 (3.9) | 106 (4.2) |
| ≥ 28 | 844 (1.4) |  | 49 (1.2) | 60 (1.3) | 16 (1.6) | 231 (1.5) | 84 (1.7) | 62 (1.0) | 48 (1.4) | 119 (1.3) | 70 (1.6) | 59 (1.3) | 46 (1.8) |

| **Supplementary Table 2 (continued) :** | | | | | | | | | | | | | |
| --- | --- | --- | --- | --- | --- | --- | --- | --- | --- | --- | --- | --- | --- |
|  |  |  | NORDCAN group | | | | | | | | | | |
|  | All cancers |  | Upper gastro-intestinal | Colorectal | Lung | Breast | Gynaeco-logical | Male genitals | Kidney & bladder | Melanoma skin | CNS | Blood | Other |
| Excluded due to: n (%**) |  |  |  |  |  |  |  |  |  |  |  |  |  |
| Disability pension | 13,204 (11.8) |  | 1,992 (16.6) | 1,324 (13.3) | 2,070 (24.8) | 2,109 (9.0) | 1,007 (12.3) | 583 (5.9) | 660 (10.3) | 291 (2.4) | 1,495 (18.3) | 987 (12.2) | 686 (13.0) |
| Age-related pension | 11,523 (10.3) |  | 768 (6.4) | 1,346 (13.5) | 385 (0.5) | 3,116 (13.2) | 840 (10.2) | 1,611 (16.3) | 958 (15.0) | 852 (7.2) | 461 (5.6) | 834 (10.3) | 352 (6.7) |
| Death | 20,009 (17.9) |  | 4,538 (37.9) | 2,222 (22.3) | 4,756 (56.9) | 1,393 (5.9) | 1,063 (12.9) | 538 (5.4) | 1,024 (16.0) | 570 (4.8) | 1,260 (15.4) | 1,176 (14.6) | 1,469 (27.9) |
| Emigration | 1,390 (1.2) |  | 130 (1.1) | 64 (0.6) | 36 (0.4) | 221 (0.9) | 110 (1.3) | 192 (1.9) | 75 (1.2) | 214 (1.8) | 156 (1.9) | 124 (1.5) | 68 (1.3) |
| Other/unknown | 4,470 (4.0) |  | 382 (3.2) | 462 (4.6) | 105 (1.3) | 950 (4.0) | 289 (3.5) | 528 (5.3) | 254 (4.0) | 666 (5.6) | 334 (4.1) | 330 (4.1) | 170 (3.2) |

* Income groups: < -60,395, -60394 – -20,132, and -20,131 – -1 have been categorized as one group due to a small number of observations.

** Percentage of total number of cancer patients in each cancer group at baseline.
